# Shape-Related Toxicity of Titanium Dioxide Nanofibres

**DOI:** 10.1371/journal.pone.0151365

**Published:** 2016-03-21

**Authors:** Manfredi Allegri, Massimiliano G. Bianchi, Martina Chiu, Julia Varet, Anna L. Costa, Simona Ortelli, Magda Blosi, Ovidio Bussolati, Craig A. Poland, Enrico Bergamaschi

**Affiliations:** 1 Department of Biomedical, Biotechnological and Translational Sciences (S.Bi.Bi.T.), University of Parma, Parma, Italy; 2 Department of Clinical and Experimental Medicine, University of Parma, Parma, Italy; 3 Institute of Occupational Medicine (IOM), Edinburgh, United Kingdom; 4 Institute of Science and Technology for Ceramic (ISTEC-CNR), Faenza, Italy; 5 ELEGI/Colt Laboratories, MRC/University of Edinburgh Centre for Inflammation Research, Queen's Medical Research Institute, Edinburgh, United Kingdom; National Institute of Health (NIH), UNITED STATES

## Abstract

Titanium dioxide (TiO_2_) nanofibres are a novel fibrous nanomaterial with increasing applications in a variety of fields. While the biological effects of TiO_2_ nanoparticles have been extensively studied, the toxicological characterization of TiO_2_ nanofibres is far from being complete. In this study, we evaluated the toxicity of commercially available anatase TiO_2_ nanofibres using TiO_2_ nanoparticles (NP) and crocidolite asbestos as non-fibrous or fibrous benchmark materials. The evaluated endpoints were cell viability, haemolysis, macrophage activation, trans-epithelial electrical resistance (an indicator of the epithelial barrier competence), ROS production and oxidative stress as well as the morphology of exposed cells. The results showed that TiO_2_ nanofibres caused a cell-specific, dose-dependent decrease of cell viability, with larger effects on alveolar epithelial cells than on macrophages. The observed effects were comparable to those of crocidolite, while TiO_2_ NP did not decrease cell viability. TiO_2_ nanofibres were also found endowed with a marked haemolytic activity, at levels significantly higher than those observed with TiO_2_ nanoparticles or crocidolite. Moreover, TiO_2_ nanofibres and crocidolite, but not TiO_2_ nanoparticles, caused a significant decrease of the trans-epithelial electrical resistance of airway cell monolayers. SEM images demonstrated that the interaction with nanofibres and crocidolite caused cell shape perturbation with the longest fibres incompletely or not phagocytosed. The expression of several pro-inflammatory markers, such as NO production and the induction of *Nos2* and *Ptgs2*, was significantly increased by TiO_2_ nanofibres, as well as by TiO_2_ nanoparticles and crocidolite. This study indicates that TiO_2_ nanofibres had significant toxic effects and, for most endpoints with the exception of pro-inflammatory changes, are more bio-active than TiO_2_ nanoparticles, showing the relevance of shape in determining the toxicity of nanomaterials. Given that several toxic effects of TiO_2_ nanofibres appear comparable to those observed with crocidolite, the possibility that they exert length dependent toxicity *in vivo* seems worthy of further investigation.

## Introduction

High aspect ratio nanostructures (HARN), such as nanotubes, nanofibres, nanowires, are increasingly used in many industrial applications from electronics to photovoltaics. However, while nanofibres continue to show their utility in many applications, the morphological similarity of these materials to pathogenic fibres, such as asbestos, has raised serious concerns about the potential health implications of exposure.

This association, based on morphology, is not simply an arbitrary link between very different materials but relies upon the structure-activity relationship that has been identified to promote fibre-type pathogenicity, as opposed to particle toxicity mediated by other mechanisms such as surface reactivity [1] or release of cytotoxic ions [2]. This structure-activity relationship, known as the “fibre pathogenicity paradigm” (FPP), identifies three critical features that are required for a fibrous particle to present a fibre-type health hazard: aspect ratio and length (dimension/shape), persistence of a particle in the biological environment and its resistance to breakage and dissolution (durability) and, most crucially for consideration of risk, the exposure to the particle in question (dose) [3]. These components of dimension, durability and dose, or the 3 Ds, provide the cornerstone of the FPP and have recently been reviewed alongside other determinants of particle induced toxicity [4,5,6,7].

Nanostructured TiO_2_ materials are among the most abundant nanomaterials produced in industrial processes and exploited in widely used products. In particular, the biological activity of TiO_2_ nanoparticles (NP), yearly produced in tonnage quantities and present in many products of common use, has been extensively characterized. Although considered low-toxicity nanomaterials, owing to their large surface area TiO_2_ NP are known to be endowed with a certain degree of cytotoxicity and to cause inflammation *in vivo* [8]. Much less is known about the toxic effects of other, more novel nanostructured TiO_2_ materials, such as nanobelts, nanotubes and nanowires [9,10,11], although some reports indicate that these materials are more toxic than NP [12,13,14]. In particular, TiO_2_ nanofibres are increasingly used as photocatalytic components in solar cells (anode in dye-sensitized solar cells), catalysts, cosmetic ingredients, preparing the ground for possible exposures, especially in occupational scenarios.

In the present study we evaluated *in vitro* the biological effects of TiO_2_ nanofibres (NF) of industrial origin, investigating in different cell models their cytotoxic and pro-inflammatory effects as well as their capability to impair epithelial monolayers. The biological effects of TiO_2_ NF have been compared with those of crocidolite, a form of asbestos, and TiO_2_ NP, used as fibrous and non-fibrous benchmarks, respectively.

## Materials and Methods

### Preparation and dispersion of materials

Nanofibres of titanium dioxide (TiO_2_ NF), produced by an electro-spinning process, were obtained from Elmarco s.r.o. (Liberec, Czech Republic), and Aeroxide® P25 TiO_2_ nanoparticles (TiO_2_ NP, 83% anatase, 17% rutile) from Evonik Degussa GmbH (Essen, Germany). UICC Asbestos crocidolite was a generous gift of Prof. Bice Fubini, University of Turin.

Powders were suspended in a sterile-filtered solution of 0.05% Bovine Serum Albumin (BSA, Sigma Aldrich, Milan, Italy) in Phosphate Buffered Saline without calcium and magnesium (PBS) to obtain 10X stock suspensions of the highest concentration tested (1.28 mg/mL, corresponding to 80 μg/cm^2^ for cells seeded in 96- and 24-well plates, or 1 mg/mL, corresponding to 80 μg/cm^2^ for cells seeded into cell culture inserts with membrane filters for 24-well multi-trays). After vortex mixing (30 sec) and water bath sonication (10 min), the stock suspensions were subsequently diluted in the same solvent to obtain 10X stocks of the other doses.

### Endotoxin (LPS) contamination analysis

Materials were suspended in endotoxin-free water at 1 mg/mL and sonicated for 10 min at room temperature (RT) in a bath sonicator (Ultrawave Sonicator QS25, 400W). After 24h incubation in endotoxin free water, samples were centrifuged at 15000g for 15 min. Supernatants were assessed for endotoxin contamination using the Pyrogen Assay (Lonza, Blackley, UK) according to the manufacturer’s instructions. Endotoxin levels were calculated according to the standard curve obtained, and results were expressed as endotoxin contamination (EU/mL).

### Characterization of TiO_2_ NF

The investigation of the fibre size distribution was performed by scanning electron microscopy using FE-SEM (Carl Zeiss Sigma NTS Gmbh, Öberkochen, Germany). Nanofibres were dispersed on a standard aluminum support by simple drop casting of the suspensions. Samples were left to air dry in a dust free atmosphere then placed on a hot-plate at 100°C for 5 min to ensure the complete evaporation of water before FE-SEM analysis. Average diameters and lengths of TiO_2_ NF samples were calculated on more than 100 nanofibres, by analyzing different images (ImageJ, Wayne Rasband, 1997). The specific surface area (SSA) measurements were determined with the Brunauer-Emmett-Teller (BET) method, using N_2_ as adsorptive gas (Sorpty 1750, Carlo Erba, Milan, Italy).

The zeta potential values of TiO_2_ NF were evaluated with a Zetasizer nano ZSP (model ZEN5600, Malvern Instruments, UK) in 0.9% NaCl. Zeta potential data were obtained by electrophoretic light scattering (ELS), and the Smoluchowski approximation was applied to convert the electrophoretic mobility to Zeta potential. Zeta potential measurements were performed on 700 μL of a NF dispersion (1 g/L) at 25°C, and the measurement duration was set to automatic as well as the attenuator position and the applied voltage. The zeta potential data were obtained by averaging three measurements.

The XRD pattern of TiO_2_ NF was obtained using a Bragg-Brentano diffractometer (Bruker D8 Advance, Karlsruhe, Germany) operating in a θ/2θ configuration, with an X-Celeretor detector LynkEye (15°–75°, 2θ range, 0.02 step size, 1 s per step).

### Cell culture

Cell analysis was performed on the mouse peritoneal monocyte-macrophage cell line Raw 264.7 and the human alveolar carcinoma epithelial cell line A549, both of which were obtained from the Cell Bank of the IZSLER (Istituto Zooprofilattico Sperimentale della Lombardia e dell’Emilia-Romagna, Brescia, Italy). Raw 264.7 cells were cultured in Dulbecco’s modified Eagle’s medium (DMEM) supplemented with 10% foetal bovine serum (FBS), 4 mM glutamine, and antibiotics (streptomycin 100 μg/mL/ penicillin, 100 U/mL), while A549 cells were cultured in F-12 Ham’s medium supplemented with 10% FBS, 1 mM glutamine, and antibiotics.

For SEM analysis (performed by a separate partner), Raw 264.7 cells were obtained from the European Collection of Cell Cultures (ECACC, Salisbury, UK) and cultured in phenol red-free Roswell Park Memorial Institute (RPMI) medium with 10% heat-inactivated FBS, 2 mM L-glutamine, and antibiotics.

Calu-3 cells, obtained from a human lung adenocarcinoma and derived from serous cells of proximal bronchial airways [15], were obtained from the IZSLER Cell Bank. Calu-3 cells were cultured in EMEM supplemented with 10% FBS, 2 mM glutamine, 1 mM sodium pyruvate, and antibiotics. All cultures were maintained in a humidified atmosphere of 5% CO_2_ in air in 10-cm dishes.

### Electron Paramagnetic Resonance (EPR)

The ability of the materials to generate Reactive Oxygen Species (ROS) was assessed in an acellular system using Electron Paramagnetic Resonance (EPR). Materials were suspended at 1 mg/mL in PBS and incubated with the spin trap 1-hydroxyl-2,2,6,6-tetramethyl-4-oxo-piperidine (Tempone-H; 1 mM) immediately before the initial measurement. Tempone-H is a highly sensitive spin trap that shows selectivity for the superoxide anion, as well as for peroxyl radicals and peroxynitrite, forming a stable product that can be measured by EPR [16]. Samples were maintained at 37°C throughout the incubation and measurements were taken after 1 and 60 min after the addition of the Tempone-H by drawing 50 μL of sample into a capillary tube (Scientific Laboratory Ltd., Coatbridge, UK) and sealing it with a plug of soft sealant (Cristaseal, VWR International, Lutterworth, UK). An X-band EPR spectrometer (Magnettech MS-200, Berlin, Germany) was used with the following parameters: microwave frequency, 9.3–9.55 Hz; microwave power, 20 mW; modulation frequency, 100 kHz; modulation amplitude, 1,500 mG; centre field, 3,365 G; sweep width, 50 G; sweep time, 30 sec; number of passes, 1. Measurement of intrinsic ROS production was conducted across 3 independent experiments and the mean signal intensity (area under the curve) established across the replicate experiments. Pyrogallol (100 μM) was used as a positive control to generate superoxide radicals [17].

### DCFH-DA assay

The membrane permeable DCFH-DA probe was used to measure the ability of materials to induce oxidative stress in cells [18,19]. After internalization, intracellular esterases cleave the diacetate moiety, thus causing probe retention and making it sensitive to ROS. DCFH was determined fluorometrically in cell lysates according to a previously described procedure [18,19], with minor modifications. Cells were seeded in 96-well plates as previously described and treated for 24h with cell culture medium with or without the materials at sub-lethal concentrations (10, 20 and 40 μg/cm^2^). After being washed twice in sodium chloride (0.9%), cells were incubated for 1h at room temperature in a solution of DCFH-DA (10 μM in sodium chloride) to allow internalization of the probe into the cell cytoplasm. Cells were then washed with sodium chloride and lysed in 90% DMSO in PBS. Plates were centrifuged at 300*g* for 15 min to remove cellular debris and particulates. The fluorescence was measured in the supernatants (λ_ex_ 485 nm; λ_em_ 530 nm) using a plate reader (Fluostar Optima, BMG Labtech, Aylesbury, UK). Results are expressed as change in RFU compared to untreated control. Using the same procedure, cells were prepared without the probe to check the material interference.

### Glutathione assay

Cells were seeded in 6-well plates and treated with medium with or without the various materials tested at sub-lethal concentrations (10, 20 and 40 μg/cm^2^) for 6h. Cells were washed in ice-cold PBS and then lysed in 200 μL ice-cold 5% trichloroacetic acid in 20 mM HCl, 2.15 mM EDTA and 10 mM ascorbic acid (all reagents from Sigma-Aldrich, Poole, UK). Samples were then transferred to Eppendorf tubes and centrifuged at 15000*g* for 15 min at 4°C. 10 μL of each sample (in duplicate) were added to 19 μL of extraction buffer in a 96-well plate. 48 μL of 1 M potassium phosphate (pH 7) were added. After 5 min of incubation, 200 μL of 0.1M potassium phosphate (pH 6.9) were added. Finally, 29 μL per well of *o*-phtalaldehyde (OPT, 5 mg/mL) in methanol was added as the OPT reacts with GSH to form a highly fluorescent product. After 30 min incubation, fluorescence (λ_ex_ 350 nm; λ_em_ 420 nm) was measured using a plate fluorimeter (Fluostar Optima, BMG Labtech, Aylesbury, UK). Reduced glutathione concentration was calculated according to standards and results were expressed as GSH level compared to untreated cell control.

### Lipid peroxidation assay (TBARs assay)

The lipid peroxidation product Malondialdehyde (MDA) was quantified as Thiobarbituric Acid Reactive Substances (TBARs). Cells, seeded in 6-well plates and treated with the materials for 24h, were lysed and samples acidified before being tested using the TBARs Parameter kit (R&D System, Abingdon, UK) according to manufacturer’s instructions. Results were calculated according to absorbance (530–540 nm) readings obtained with standards and expressed as TBARs (μM).

### Cell viability

For cell viability analysis, cells were seeded in complete growth medium at a density of 9.3x10^4^ cells/cm^2^ (Raw 264.7) or 3.1x10^4^ cells/cm^2^ (A549) in 96-well Microtest™ plates (Falcon, Corning Inc., Corning, NY, USA). 24h after cell seeding, growth medium was replaced with fresh medium supplemented with the materials (dose range 2.5–80 μg/cm^2^). In all the experiments, the vehicle (0.05% BSA in PBS) was added to the control at the minimal dilution used.

Cell viability was assessed with resazurin assay. In the resazurin assay, a non-fluorescent, membrane permeant molecule is converted by intracellular enzymes in the fluorescent compound resorufin (λ_em_ = 572 nm) [20]. After 24, 48 and 72h of incubation with the materials, cell viability was tested replacing medium with a solution of resazurin (44 μM) in serum-free medium. After 20 min, fluorescence was measured at 572 nm with a multimode plate reader Enspire (Perkin Elmer Waltham, MA, USA). To exclude possible interference on the test by the nanomaterials, the dye was incubated with materials only (80 μg/cm^2^), and fluorescence measured. No fluorescence signal was detected above the background.

### Haemolytic activity

Haemolytic activity is a parameter suggestive of cytotoxic activity *in vivo* [21]. The assay was performed following previously described procedures [22,23] with minor modifications. Briefly, the test samples were suspended in sodium chloride 0.9% at a stock concentration of 6 mg/mL and prepared as previously described. An aliquot of sheep blood in Alsever’s solution liquid (Fisher Scientific, Loughborough, UK) was centrifuged at 250*g* for 10 min and then washed 3 times in sodium chloride 0.9%. After centrifugation, the supernatant was discarded, and 200 μL of packed erythrocytes were diluted into 7.2 mL of sodium chloride. One hundred and fifty μL per well of saline with or without the materials were added in a clear 96-well plate. Then 75 μL per well of the erythrocyte suspension were added giving a final concentrations of each material of 4, 2, 1 mg/mL. The plates were incubated for 15 min at room temperature protected from light on a plate shaker and then centrifuged at 250*g* for 15 min to pellet erythrocytes and materials. 100 μL of supernatant were then transferred into a new clear 96-well plate, and the absorbance was read at 540 nm with a plate reader (Fluostar Optima, BMG Labtech, Aylesbury, UK). Results were expressed as percentage haemolysis, with 0% being set for the saline control and 100% set for Triton X100 0.1% in sodium chloride, used as a positive control.

### Measurement of the Trans-Epithelial Electrical Resistance

For these experiments, Calu-3 cells were seeded into cell culture inserts with membrane filters (pore size 0.4 μm) for Falcon 24-well-multitrays (Becton, Dickinson & Company, Franklin Lakes, NJ, USA) at a density of 2.3 × 10^4^ cells/cm^2^, and grown for 12d until a tight monolayer was formed, as demonstrated by the high values of Trans-Epithelial Electrical Resistance (TEER > 1000 Ω·cm^2^). Materials were then added in the apical chamber from a 1 mg/mL stock solution without changing the medium, and TEER measured at the indicated times of treatment. TEER changes were expressed as the percentage of the initial value adjusted for control cell monolayers according to Eq 1 [24]:

$$\%\;\Delta TEER=\frac{Final\;TEER\;\left(treated\right)}{Final\;TEER\;\left(control\right)}\;\text{x}\;\frac{Initial\;TEER\;\left(control\right)}{Initial\;TEER\;\left(treated\right)}\;\text{x}\;100$$ (1)

### Nitrite medium concentration

Nitrite concentration, as a proxy for NO production, was determined through a fluorimetric approach, based on the production of the fluorescent molecule 1*H*-naphthotriazole from 2,3-diaminonaphthalene (DAN) in an acid environment [25]. After the selected period of incubation with the materials, 100 μl of medium were transferred to black 96-well plates with a clear bottom (Corning, Corning, NY, USA). DAN (20 μl of a solution of 0.025 mg/mL in 0.31 M HCl) was then added and, after 10 min at RT, the reaction was stopped with 20 μl of 0.7 M NaOH. Standards were performed in the same medium from a solution of 1 mM sodium nitrite. Fluorescence (λ_ex_ 360 nm; λ_em_ 430 nm) was determined with a multimode plate reader Perkin Elmer Enspire (Perkin Elmer, Waltham, MA, USA).

### Real Time-PCR

Total RNA was isolated with GenElute Mammalian Total RNA Miniprep Kit (Sigma–Aldrich). After reverse transcription, aliquots of cDNA from each sample were amplified in a total volume of 25 μl with Go Taq PCR Master Mix (Promega Italia, Milan, Italy), along with the forward and reverse primers (5 pmol each) reported in Table 1. Real time PCR was performed in a 36-well RotorGeneTM3000, version 5.0.60 (Corbett Research, Mortlake, Australia). For all the messengers to be quantified, each cycle consisted of a denaturation step at 95°C for 30s, followed by separate annealing (30s) and extension (30s) steps at a temperature characteristic for each pair of primers (Table 1). Fluorescence was monitored at the end of each extension step. At the end of the amplification cycles a melting curve analysis was added. Data analysis was made according to the relative standard curve method [26]. RT-PCR data were expressed as the ratio between each investigated mRNA and *Gapdh* mRNA.

**Table 1:** Primers and temperatures of annealing adopted for RT-PCR experiments.

Gene	Protein	Forward	Reverse	T (°C)	Amplicon Size (bp)
*Nos2*	Inducible Nitric oxide synthase (Nos2)	5'-GTT CTC AGC CCA ACA ATA CAA GA-3'	5'-GTG GAC GGG TCG ATG TCA C-3'	57°C	127
*Ptgs2*	Cyclooxygenase-2 (Cox2)	5’-GCTCAGCCAGGCAGCAAATC-3’	5’-ATCCAGTCCGGGTACAGTCA-3’	56°C	107
*Hmox1*	Hemeoxygenase-1 (HO-1)	5'-TGT TCC TAC CCC CAA TGT GT-3'	5'-GGT CCT CAG TGT AGC CCA AG-3'	57°C	137
*Gapdh*	Glyceraldehyde 3-phosphate dehydrogenase	5'-TGT TCC TAC CCC CAA TGT GT-3'	5'-GGT CCT CAG TGT AGC CCA AG-3'	57°C	137

### Scanning Electron Microscopy (SEM)

Cells were seeded on coverslips in 24-well plates (ThermanoxTM, Scientific Laboratory Supplies Limited, Hessle, UK). After 24h, cells were treated with cell culture medium with or without the various test samples at a concentration of 10 μg/cm^2^. After 24h, cells were rinsed in sodium chloride and fixed overnight at 4°C in glutaraldehyde (3% in 0.1 M sodium cacodylate buffer). The preparations were gradually dehydrated with subsequent incubation with ethanol 75%, 85%, 95% and 100%. Coverslips were then treated for 10 min with hexamethyldisilazane and, after drying, mounted on SEM stubs. Samples were sputter coated with gold using a S150B Sputter Coater (Edwards, UK) and analyzed using a Hitachi S-2600N digital Scanning Electron Microscope (SEM) fitted with an X-Max detector (50 mm^2^) for Energy Dispersive Spectroscopy (EDS) (Oxford Instruments, UK). The microscope was operated with an accelerating voltage of 20kV.

### Confocal microscopy

Confocal microscopy was performed as previously described with minor modifications [27]. Cells were seeded on four-chamber slides at a density of 15 × 10^4^ cells/cm^2^ and treated after 24h with TiO_2_ NF at the dose of 10 μg/cm^2^. The incubation was prolonged for 24h. 20 min before the end of exposure, cells were transferred in serum-free medium supplemented with CellTracker™ Red CMPTX (8 μM, Molecular Probes, Invitrogen, Carlsbad, CA, USA) to label cytoplasm; in the last 5 min 1,5-bis[2-(di-methylamino)ethyl]amino-4,8-dihydroxyanthracene-9,10-dione (DRAQ5^®^, 20 μM, Alexis Biochemicals, San Diego, CA, USA) was also added to the incubation medium to counterstain nuclei.

Confocal analysis was carried out with a LSM 510 Meta scan head integrated with an inverted microscope (Carl Zeiss, Jena, Germany). Samples were observed through a 63x (1.4 NA) oil objective. Image acquisition was carried out in multitrack mode, i.e. through consecutive and independent optical pathways. Excitation at 488 nm and reflectance were used to visualize TiO_2_ NF; excitation at 543 nm and emission recorded through a 580–630 nm band pass barrier filter were used to visualize the cytoplasm; excitation at 633 nm and emission through a 670 nm long pass filter were adopted to visualize the nucleus.

### Chemicals

Sigma-Aldrich was the source of all the chemicals whenever not stated otherwise.

### Statistics

All experiments were performed a minimum of two times, each at least in duplicate. Results were expressed as mean +/- Standard Error of the Mean (SEM) or Standard Deviation (SD), as indicated. Statistical analysis was performed by ANOVA followed by Bonferroni post hoc test. GraphPad Prism ™ software version 6.00 (GraphPad Software Inc., San Diego, CA) was used. The differences were considered significant if p<0.05.

## Results

### Characterization of TiO_2_ nanofibres

**Fig 1:**
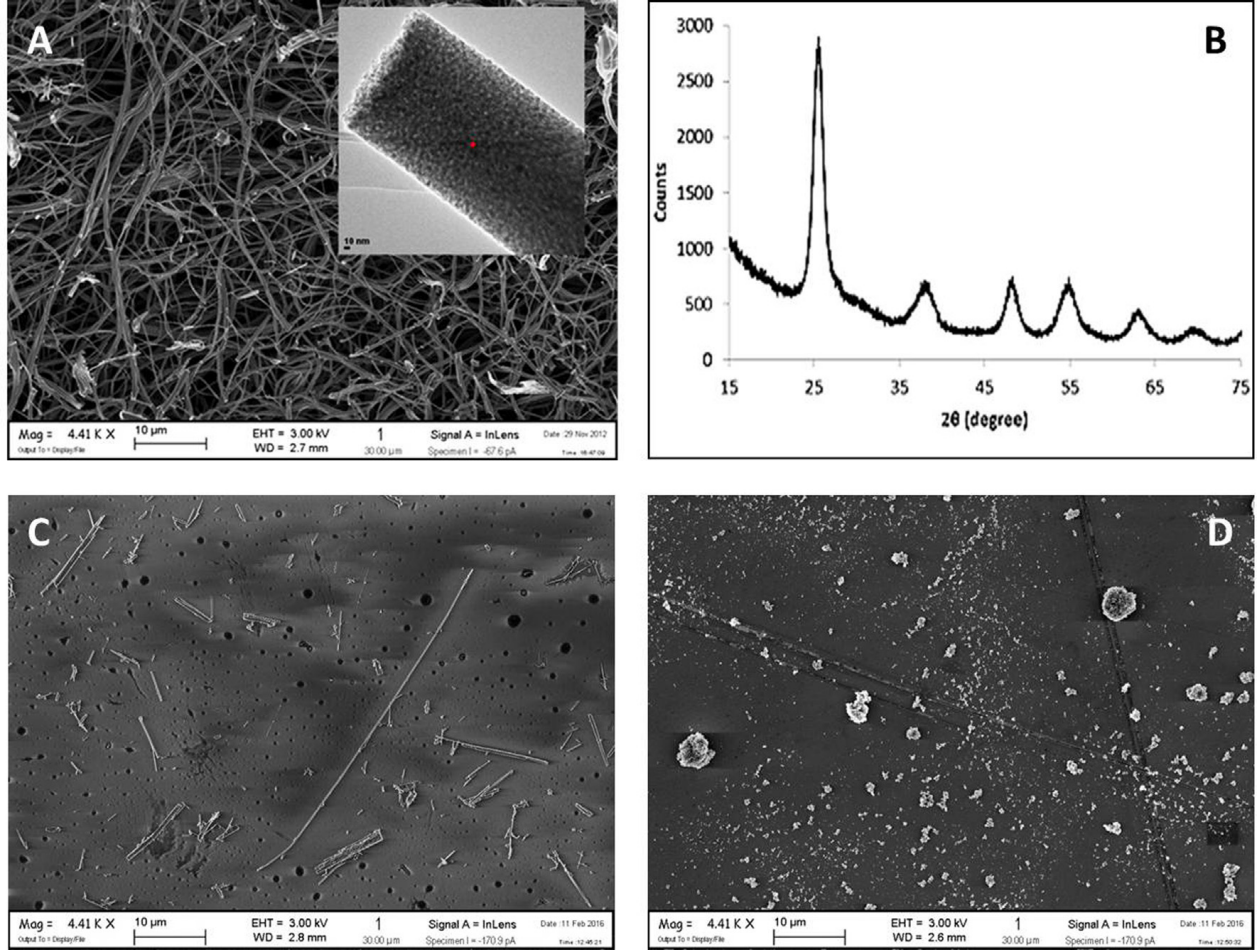
FE-SEM image and XRD pattern of TiO_2_ nanofibres. **(A)** The sample of TiO_2_ NF consists of discrete units of primary nano-particles, evident in the inset at higher magnification. **(B)** The XRD pattern of the material. **(C)** FE-SEM image of crocidolite. **(D)** FE-SEM image of TiO_2_ NP. For **(A)**, **(C)** and **(D)**. Bars, 10 μm.

The characteristics of TiO_2_ NF are recounted in Table 2. The materials was found to be very inhomogeneous (diameters ranging from about 200 to 1000 nm) but showed the desired nanostructured morphology, as demonstrated by high specific surface area (SSA; 91.2 m^2^/g, BET) and by FE-SEM imaging (Fig 1A). For comparison, images of crocidolite (Fig 1C) and TiO_2_ NP (Fig 1D) are also shown. Anatase was the crystalline phase of TiO_2_ NF, as demonstrated by XRD analysis (Fig 1B).

**Table 2:** Properties of TiO_2_ NF and benchmark materials.

Sample	Average Length (μm)	Average Diameter (μm)	Average AR^a^	Zeta Potential (mV)^b^	Crystallinity
TiO_2_ NF	9.9 ± 5.8	0.3 ± 0.1	29:1	16.0 ± 0.6	Anatase^c^
TiO_2_ NP	-	21^d^	-	13.3 ± 1.4	Anatase 83/Rutile 17^e^
UICC Crocidolite^f^	2.5 ± 2.0	0.33 ± 2.1	7.5:1	ND^g^	NA^h^

^a^ AR–Aspect Ratio

^b^ 1 g/L in NaCl 0.9%

^c^ X-ray diffraction (XRD). See Fig 1B.

^d^ According to supplier specification and confirmed by Jensen et al. [28]

^e^ [29]

^f^ Data from [30]

^g^ ND—Not determined

^h^ NA–Not applicable

Analysis of the test materials for the presence of bacterial lipopolysaccharide (LPS, see Materials and methods) showed that the endotoxin level in both TiO_2_ NF and TiO_2_ NP (used as a non-fibrous benchmark material), were below the detection limit of the assay (< 0.1 EU/mL) whereas crocidolite, used as a fibrous benchmark material, showed the presence of endotoxin contamination above the upper detection limit (5 EU/mL).

### Reactive Oxygen Species (ROS) production and oxidative stress

**Fig 2:**
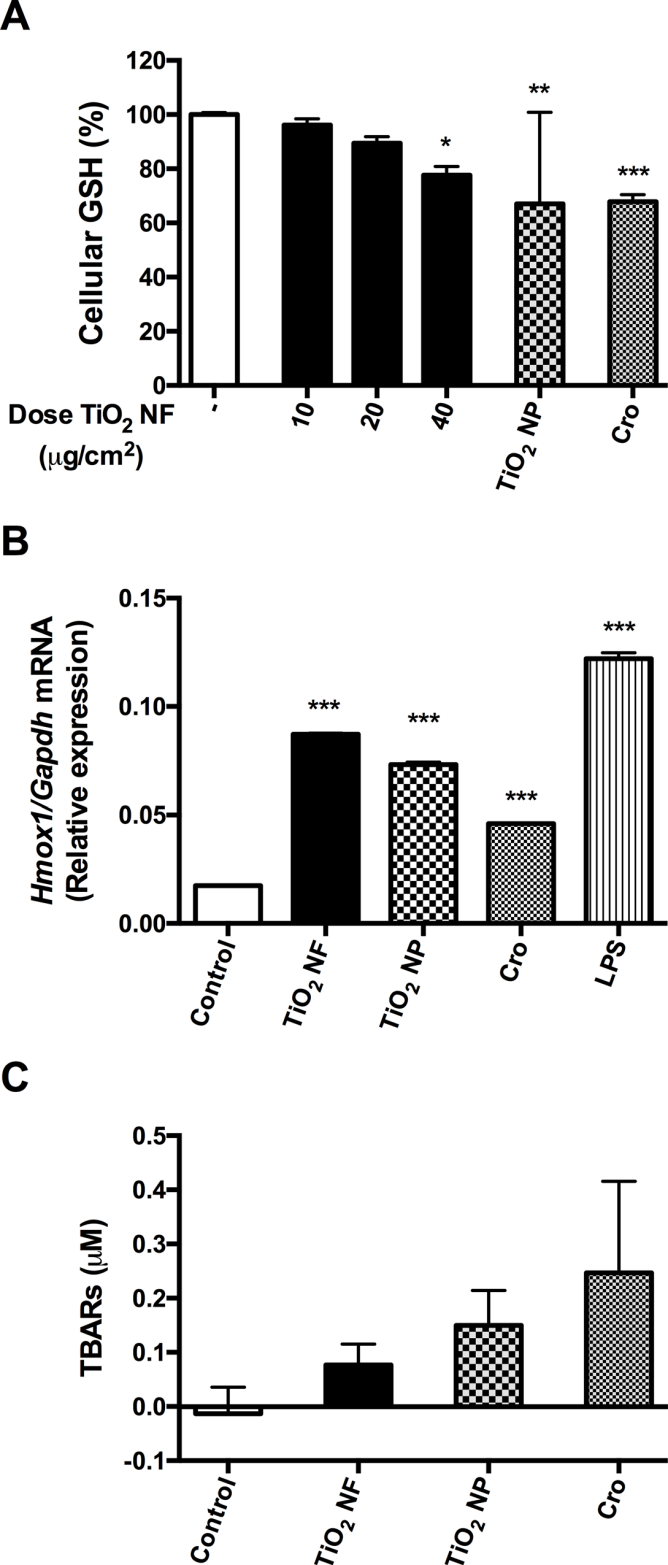
Glutathione depletion, *Hmox1* induction, and lipid peroxidation in macrophages. Glutathione (GSH) content **(A)**, the expression of *Hmox1* **(B)** and the level of thiobarbituric acid reactive substances (TBARs) **(C)** were determined in Raw 264.7 cells after a 12-hour **(B)** or 24-hour **(A-C)** incubation with or without the indicated materials. For TiO_2_ NF, the doses were 10, 20, 40 μg/cm^2^ **(A),** 80 μg/cm^2^ **(B)**, or 40 μg/cm^2^ **(C)**; for crocidolite (Cro) and TiO_2_ NP, the doses were 40 μg/cm^2^ **(A,C)** or 80 μg/cm^2^ **(B)**. Results are expressed as % of decrease **(A)** or relative expression **(B)** or fold-change **(C)** *vs*. control, untreated cultures. **(A,C)** Data are means ± SEM of 9 independent determinations. * p<0.05, ** p<0.01, *** p<0.001 *vs*. control. **(B)** Data are means ± SD of 4 values obtained in 2 separate experiments.

The intrinsic activity in terms of ROS production exhibited by the various test samples was assessed by co-incubation with the spin trap (Tempone-H) to detect the release of oxygen-centred free radicals. The results showed that both TiO_2_ NF and NP induced comparable but small ROS production, while crocidolite caused very high levels of ROS production (in excess of the positive control, consisting of 100 μM pyrogallol, data not shown). Although cell production of ROS, as assessed with the DCFH test, was not significantly increased (data not shown), all the materials caused a significant decrease in the cellular antioxidant glutathione (GSH; reduced form) within treated macrophages (Fig 2A). Consistently, *Hmox1*, the gene that encodes hemeoxygenase-1, which is known to play a pivotal role in the oxidative stress-mediated responses [31], was significantly induced in cells exposed to TiO_2_ NF with a comparable effect to that elicited by TiO_2_ NP (Fig 2B). Also crocidolite determined a significant induction of the gene although slightly lower than that observed with either TiO_2_ NP or TiO_2_ NF. None of the compounds tested induced a significant increase of lipid peroxidation in macrophages, although a trend (Crocidolite> TiO_2_ NP > TiO_2_ NF) was clearly detectable (Fig 2C).

### Cell viability and cytotoxicity

**Fig 3:**
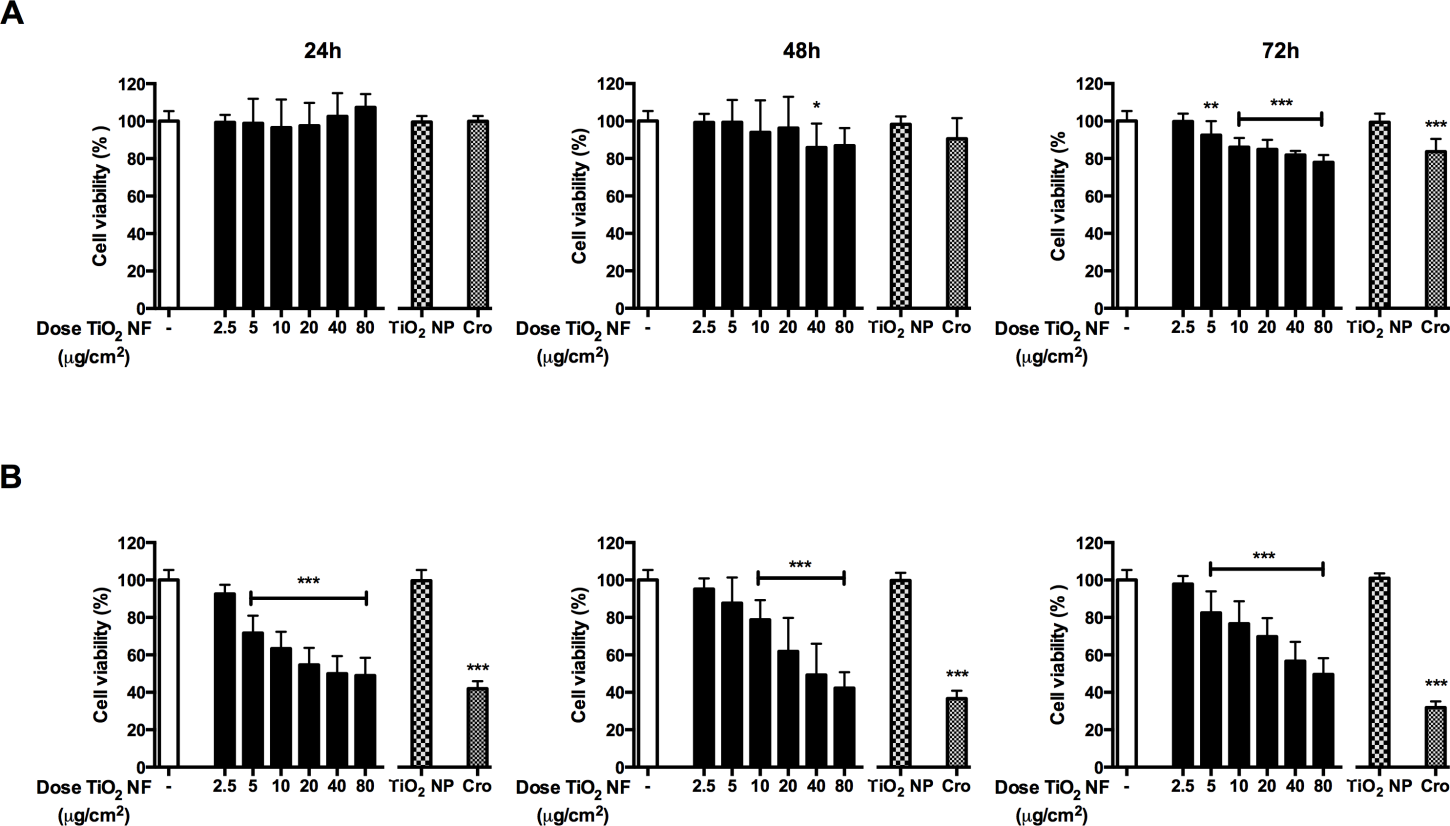
Effect of tested particles on cell viability. Raw 264.7 **(A)** and A549 cells **(B)** were treated up to 72h with or without pristine TiO_2_ NF (range dose 2.5–80 μg/cm^2^), crocidolite (Cro; 80 μg/cm^2^) or TiO_2_ NP (80 μg/cm^2^), and cell viability was assessed with the resazurin assay. Data are means ± SD of twelve independent determinations obtained in three experiments. * p < 0.05, ** p < 0.01, *** p < 0.001 *vs*. control (untreated cultures).

The effects of TiO_2_ NF (dose range 2.5–80 μg/cm^2^) on the viability of Raw 264.7 and A549 cells were tested by resazurin assay after 24, 48 and 72h-exposure (Fig 3). In macrophages (Panel A), TiO_2_ NF slightly lowered cell viability inducing a maximal decrease of 22% after 72h of incubation at the highest dose tested (80 μg/cm^2^). At this dose, the TiO_2_ NP sample, used as a non-fibrous benchmark material, did not affect cell viability, while crocidolite had a modest effect, comparable to that of TiO_2_ NF.

In A549 cells (Panel B), TiO_2_ NF significantly lowered cell viability in a dose-dependent manner with a significant reduction already detectable at 24h. The maximal decrease was detected at 48h (50%, 80 μg/cm^2^). TiO_2_ NP did not significantly affect cell viability, while crocidolite markedly lowered cell viability of A549 cells in a time-dependent manner.

To assess the membrane interaction of the materials, the samples were co-incubated with red blood cells and the level of haemolysis measured (Table 3). TiO_2_ NF was significantly more haemolytic than either TiO_2_ NP or crocidolite, with an effect larger than 40% at the maximal dose tested. The haemolytic activity of all the materials was dose-dependent.

**Table 3:** Haemolytic activity of the materials.

Sample	Dose (mg/mL)	Percentage Haemolysis (Mean)	SEM
**TiO**_**2**_ **NF**	4**	42.3	12.9
	2	23.4	4.3
	1	12.9	8.9
**TiO**_**2**_ **NP**	4	10.4	2.4
	2	2.3	0.8
	1	0.4	1.2
**CRO**	4	8.0	5.8
	2	2.9	0.7
	1	2.4	1.4

Data are means (± SEM) of three experiments, each performed in triplicate.

** p < 0.01 *vs*. equivalent doses of TiO_2_ NP and Crocidolite.

### Barrier competence of CaLu-3 monolayers

**Fig 4:**
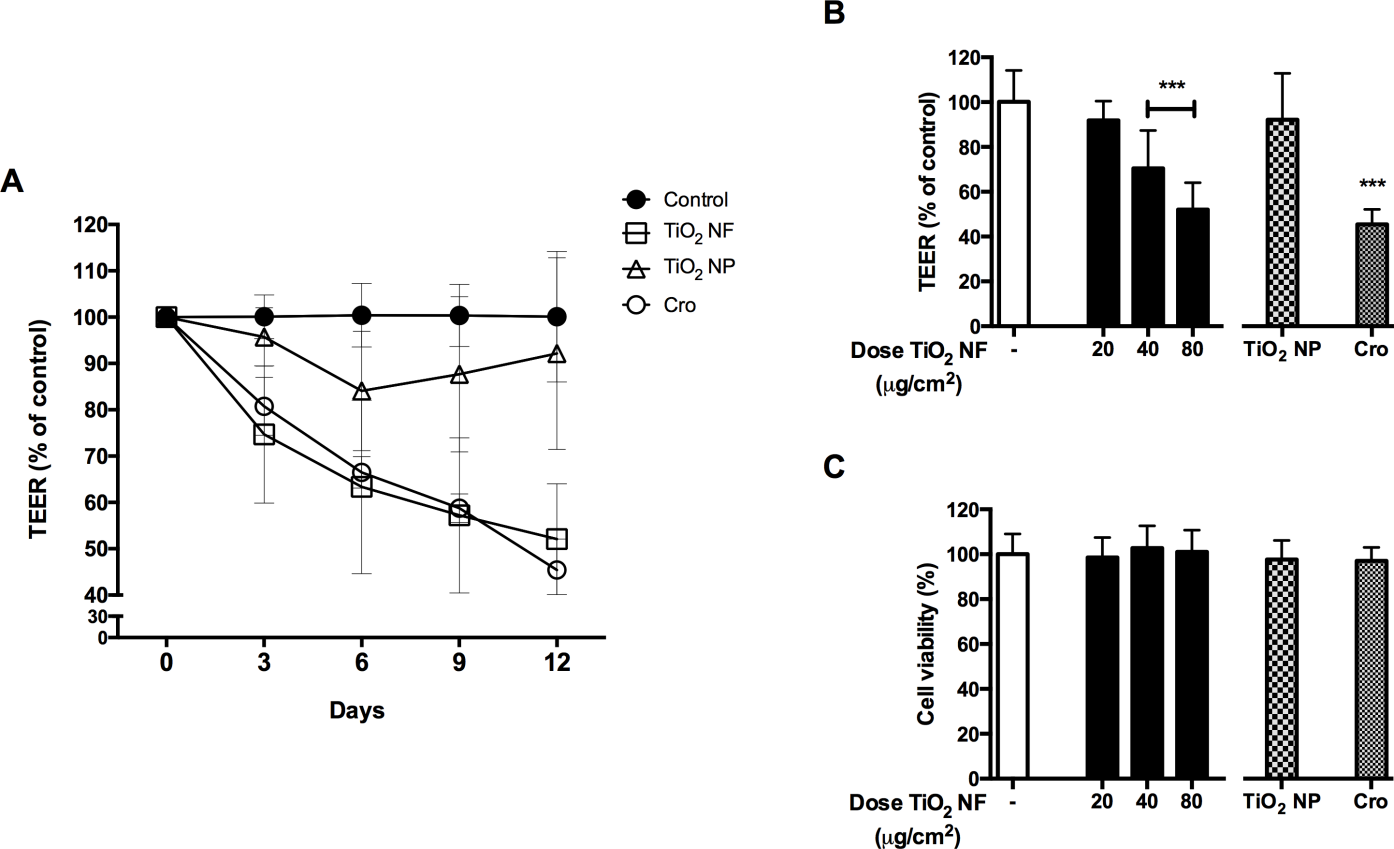
Effect of tested materials on barrier integrity and cell viability of CaLu-3 cell monolayers. The Trans-Epithelial Electrical Resistance (TEER), as a proxy of epithelial barrier competence, and cell viability were assessed in confluent monolayers of CaLu-3 cells after incubation with TiO_2_ NF, TiO_2_ NP or crocidolite (Cro) all used at 80 μg/cm^2^. **(A)** TEER (% of control) was recorded every 3d up to 12d. **(B)** TEER (% of control) measured at 12d. **(C)** Effect of TiO_2_ NF on cell viability, assessed by resazurin assay at the end of TEER experiments. Data are means ± SD of 8 independent determinations. *** p < 0.001 *vs*. control (untreated cultures).

Fig 4 (Panel A) reports the time course of changes in Trans-Epithelial Electrical Resistance (TEER) of CaLu-3 cell monolayers exposed to increasing doses of TiO_2_ NF (40–80 μg/cm^2^) up to 12d. TEER is a parameter associated with the integrity and the barrier competence of epithelial monolayers [32,33] with CaLu-3 cells here used as a model system. TiO_2_ NF significantly lowered TEER by 24% after 3d of exposure to the highest dose tested (80 μg/cm^2^), with a maximal decrease of 54% at 12d, indicating a clear dose- and time-dependent effect on barrier competence. Crocidolite (80 μg/cm^2^) produced a decrease of TEER comparable to that caused by TiO_2_ NF. On the contrary, TiO_2_ NP did not alter TEER. Fig 4 (Panel B) shows TEER changes after 12d of treatment. Cell viability, monitored at the end of the experiment in the same wells where TEER evaluation had been performed, exhibited no significant changes under any experimental condition (Fig 4, Panel C).

### Expression of pro-inflammatory markers in macrophages

**Fig 5:**
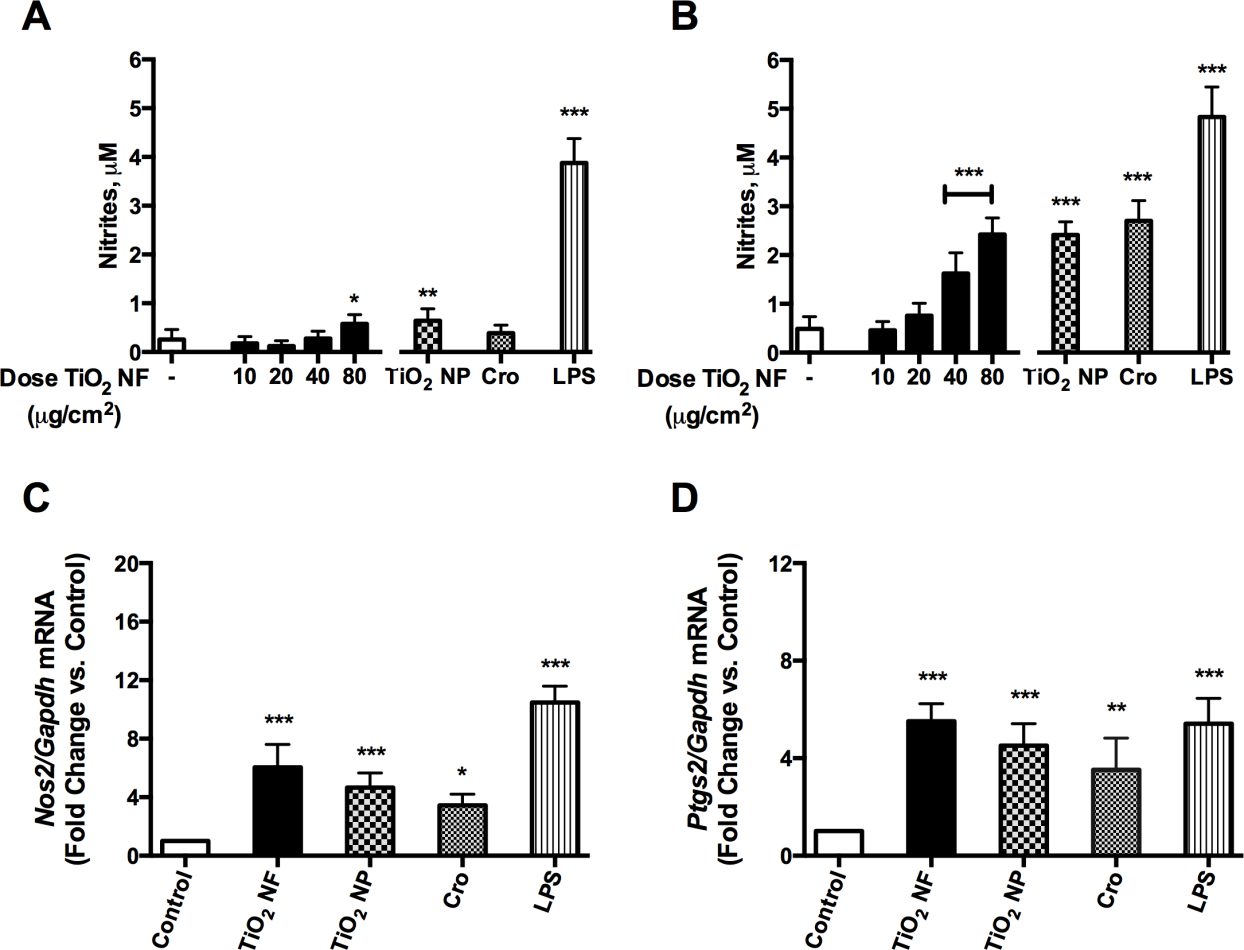
Expression of pro-inflammatory markers in macrophages. Macrophages were incubated up to 72h with TiO_2_ NF, at the indicated doses, TiO_2_ NP and crocidolite (Cro) at 80 μg/cm^2^, or with 10 ng/mL of LPS as a positive control. **(A, B)** Effects of TiO_2_ NF (dose range 10–80 μg/cm^2^) on NO production after 48h **(A)** or 72h **(B)**. **(C, D)** Effects of the indicated materials (all used at 80 μg/cm^2^) on *Nos2* **(C)** or *Ptgs2* **(D)** gene expression in Raw 264.7 cells, assessed with RT-PCR after a 24h-exposure. **(A, B)** Data are means ± SD of 9 independent determinations obtained in three experiments. **(C, D)** Data are means ± SD of 4 independent determinations obtained in two experiments. For all the panels, * p < 0.05, ** p < 0.01 *** p < 0.001 *vs*. control (untreated cultures).

Fig 5 reports nitric oxide (NO) production, as assessed from nitrite concentration in the culture medium after 48h (Panel A) or 72h (Panel B) of treatment of Raw 264.7 cells with increasing doses of TiO_2_ NF (range 10–80 μg/cm^2^). In cells treated with TiO_2_ NF, NO production was already increased at 48h compared with control (2-fold at 80 μg/cm^2^), reaching a maximal stimulation after 72h (5-fold at 80 μg/cm^2^). TiO_2_ NP and crocidolite, both used at 80 μg/cm^2^, significantly stimulated NO production after a 72h-treatment. NO production was consistently associated with the induction of *Nos2* (Fig 5C). The pro-inflammatory effect of TiO_2_ NF was not limited to *Nos2* induction and NO production, but involved also the expression of another pro-inflammatory marker, *Ptgs2* gene (encoding for the inducible form of cyclooxygenase, Cox2), which was significantly increased by TiO_2_ NF (5-fold at 80 μg/cm^2^, Fig 5D).

### Interaction of materials with macrophages

**Fig 6:**
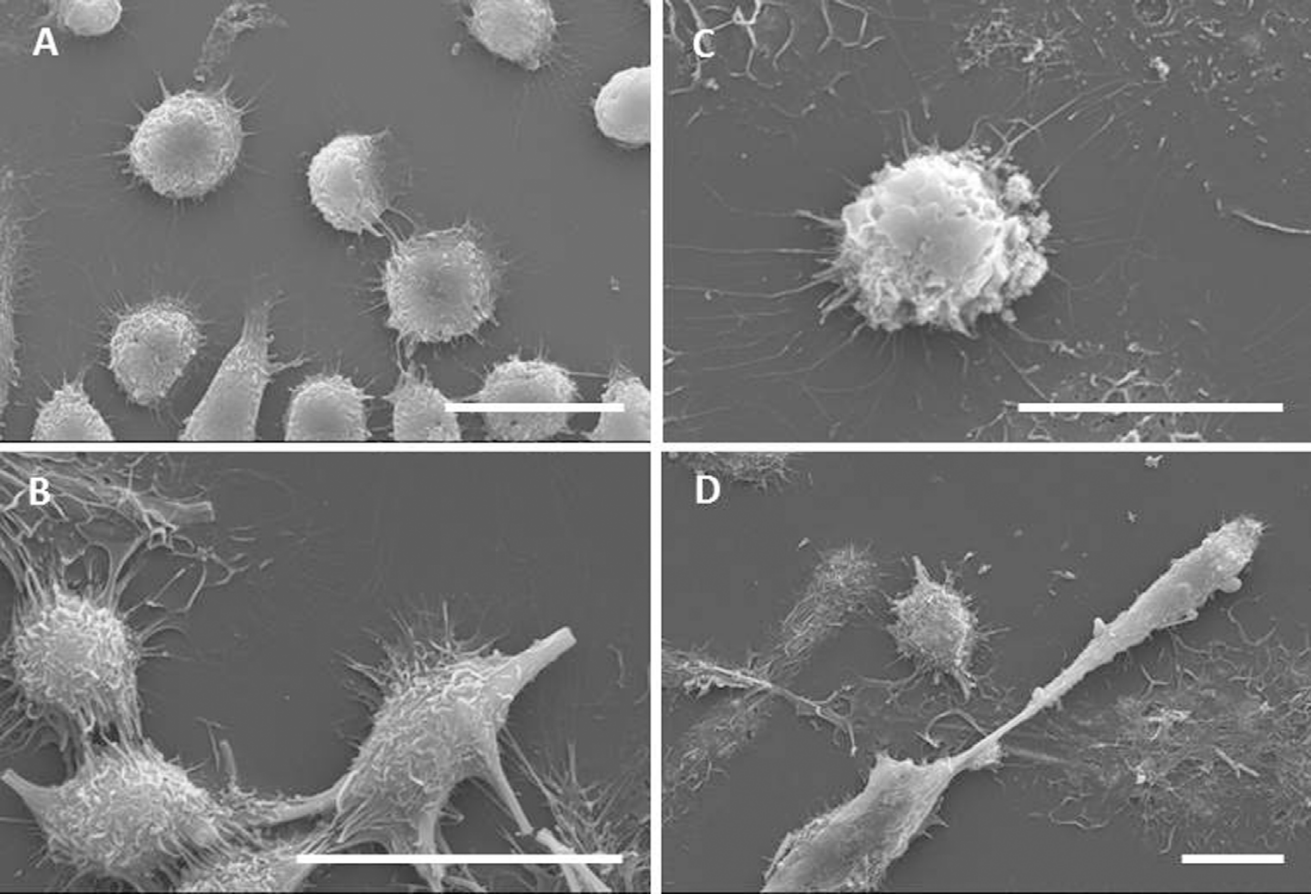
Characterization of the interaction between macrophages and materials by scanning electron microscopy. Raw 264.7 cells were seeded on coverslips and incubated for 24h in the absence **(A)** or in the presence of TiO_2_ NF **(B)**, TiO_2_ NP **(C)** or crocidolite asbestos **(D)** at a dose of 10 μg/cm^2^. Cells were then labelled, fixed, mounted for SEM analysis as described in Materials and methods. Bars, 20 μm.

In order to study how cells interact with the NF, macrophages exposed to the materials (all at the dose of 10 μg/cm^2^) were observed with scanning electron microscopy. In control, untreated cultures (Fig 6A), macrophages were mostly rounded with few elongated cells, suggesting a certain degree of spontaneous activation. On the contrary, macrophages treated with TiO_2_ NF appeared heavily stretched along the fibres (Fig 6B), with the longest fibres not fully internalized and protruding from the cells. In other cases, fibres were shared by two or more cells, indicating that macrophages were engaging with the material but were unable to engulf it completely. Similar images were detected in cells exposed to crocidolite (Fig 6D). Macrophages treated with TiO_2_ NP mostly exhibited rounded morphology, although several cells exhibited protrusions and processes (Fig 6C).

**Fig 7:**
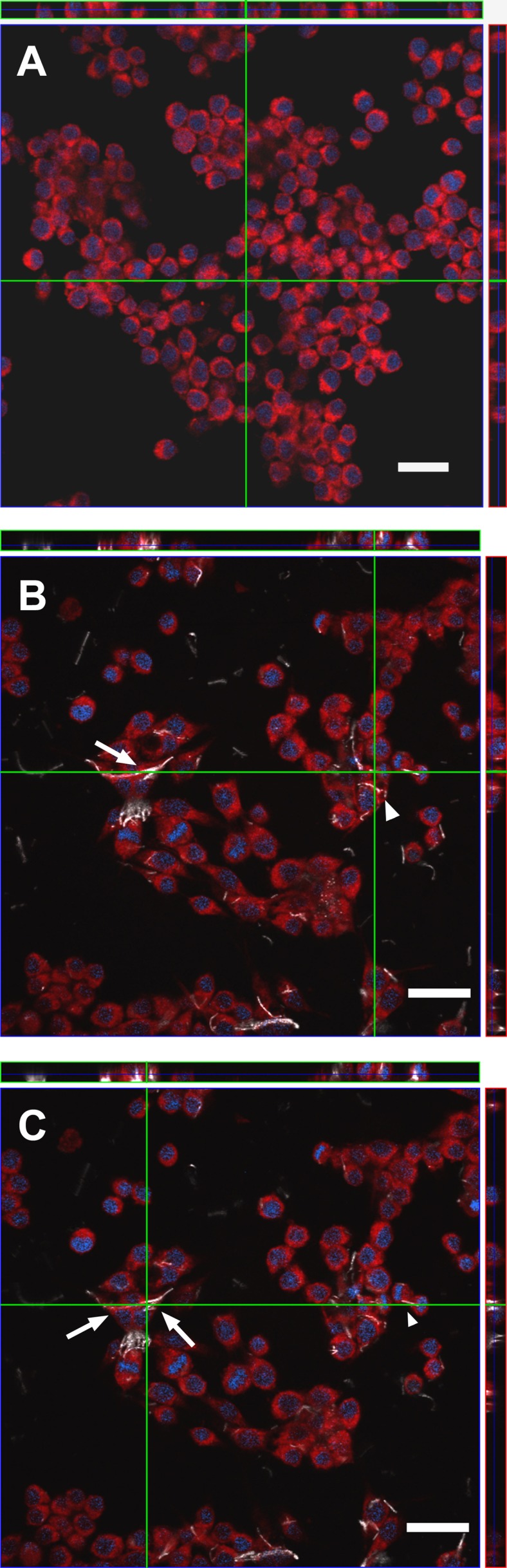
Characterization of the interaction between macrophages and TiO_2_ NF by confocal microscopy. Raw 264.7 macrophages were seeded on coverslides and incubated for 24h with TiO_2_ NF (10 μg/cm^2^). At the end of the experiment, cells were labelled and fixed as detailed in Materials and methods. **(A)** A single horizontal section of control, untreated cells is shown along with two orthogonal projections. **(B, C)** two single horizontal sections of NF-treated cells, taken at different planes, are shown along with two orthogonal projections. In **(B)** and **(C)**, arrows point to pattern suggestive of cell fusion and arrowheads to an example of internalized material. In each panel, green lines highlight the planes where the orthogonal projections were taken. White, TiO_2_ NF; Blue, nucleus; Red, cytoplasm. Images report representative fields. Bars, 20 μm.

Confocal microscopy (Fig 7) was used to investigate in depth cell morphology/behavior upon exposure to TiO_2_ NF. In cultures exposed to the material (Panels B and C), bundles of nanofibres were readily detected. The shorter bundles were internalized, as demonstrated by the orthogonal sections that show pieces of material completely surrounded by cell cytoplasm. However, no co-localization of internalized NF with the cytoplasmic marker was observed, suggesting the compartmentalization of the material. Several cells tended to adhere simultaneously to the longer bundles, spreading along their long axis. In some cases, these bundles appeared partially internalized, as in the case of the huge bundle highlighted by the arrows, which seems to partially adhere to the culture surface. In both the horizontal sections, the individual cells interacting with this bundle were not easily distinguished, a pattern compatible with the formation of a multinucleated syncytium.

## Discussion

This contribution reports the results of the toxicological characterization of an industrial preparation of TiO_2_ nanofibres (NF). The biological effects observed have been compared with those caused by a non-fibrous nanostructured material of the same chemical composition, TiO_2_ nanoparticles (NP), using crocidolite asbestos as a fibrous benchmark material.

The NF used fully cope with the dimensional criteria of a respirable fibre as defined by the WHO: a length greater than 5 μm, a diameter less than 3 μm and a length-to-width ratio (aspect ratio) greater than 3:1 [34]. These dimensional criteria dictate if the fibre is respirable based on its diameter, because the fibre aerodynamic diameter is proportional to the fibre diameter rather than to the length, and only very slightly to increased aspect ratio [35]. The minimum length requirement of 5 μm reflects the role that length has in determining fibre-type pathogenicity.

The materials were assessed *in vitro* for their toxicity towards macrophages and alveolar epithelial cells, which represent key target cells within the lung, the organ of primary concern for exposure to fibrous materials. The doses selected for the evaluation of TiO_2_ NF toxicity encompassed a large range (2.5–80 μg/cm^2^) with the primary aim of covering either the low dose range, which is of the greatest relevance to occupational exposure, or the higher dose range, where subtler differences in toxicity may become more apparent (although the relevance of such effects at high doses must always be critically evaluated). To place this dose range into context, we calculated an estimated human equivalent dose (HED) by scaling the *in vitro* dose per cm^2^ to the retained dose taking into account clearance halftime (representing the steady state equilibrium obtained during chronic exposure) based on human alveolar surface area. This was then used to calculate a deposited dose rate followed by the inhaled dose rate and, finally, an estimation of the human exposure (mg/m^3^), which would give rise to this equivalent retained dose. The deposited dose rate was based on a deposition efficiency of 9% in the alveolar region in humans, calculated using the MPPD (V2.11) software and based on published particle aerodynamic parameters. Specifically, these parameters were a mass median aerodynamic diameter (MMAD) of 0.8 μm, a geometric standard deviation (GSD) of 1.4 μm at an aerosol concentration of 33 mg/m^3^ [36], and a true density by helium pycnometry of 4.2213 g/cm^3^ [37,38]. Using this approach, we estimate that the human equivalent (chronic) exposure at the low *in vitro* doses are high, but plausible levels of 7.3, 14.7 and 29.3 mg/m^3^ for *in vitro* doses of 2.5, 5 and 10 μg/cm^2^, respectively.

The evaluation of cell viability of the exposed cultures indicated that TiO_2_ NF caused a moderate decrease in cell viability, comparable to that observed with crocidolite, while TiO_2_ NP were ineffective even at the high dose tested (80 μg/cm^2^). However, the particle surface area is higher for TiO_2_ NF (91.2 m^2^/g) than for the benchmark NP (53 m^2^/g [29]), raising the possibility that the different activities of NF and NP may reflect a dosimetric issue. This is not the case. Indeed, if one considers the results reported in Fig 3, it is evident that significant effects on cell viability are observed at doses much lower, when expressed as m^2^/cm^2^, than those reached with 80 μg/cm^2^ of TiO_2_ NP. For example, at 10 μg/cm^2^, the dose of NF, expressed as specific area, would be 0.00091 m^2^/cm^2^ compared with 0.00424 m^2^/cm^2^ obtained with 80 μg/cm^2^ of TiO_2_ NP. These considerations indicate that shape, rather than chemical composition, play the the prevailing role in determining toxicity. The calculated IC_20_, taken as the toxic threshold effect concentration, was 6.1 μg/cm^2^ for macrophages and 5 μg/cm^2^ for alveolar epithelial cells. The difference in sensitivity to NF between macrophages and epithelial cells may reflect the different specialized function typical of these cell types: a relatively passive epithelial cell, in terms of particle interaction, as opposed to macrophages, which actively seek out and engulf particles thereby accumulating dose. [29]

Similar effects of crocidolite and TiO_2_ NF were also noted as far as TEER decrease, an indicator of epithelial barrier perturbation, is concerned. While changes in TEER have been described in the past for chrysotile [39,40,41,42], this is, to our knowledge, the first report in which barrier perturbing effects are described for crocidolite. However, also in this case, TiO_2_ NP were ineffective, indicating the prevailing role of shape as toxicity determinant. Loss of epithelial barrier competence and monolayer integrity are important parameters correlated to penetration of the nanomaterials in the bronchial wall, alterations of epithelial functions, and nanomaterial bio-persistence [33]. A comparable effect was observed in the same cell model upon exposure to long, needle-like [43] but not to shorter Multi-walled Carbon Nanotubes (MWCNT) [44]. In the case of MWCNT, loss of barrier integrity has been attributed to focal damage of the epithelial cell monolayer, due to the interaction with nanomaterial agglomerates. Although this issue has not been specifically addressed here, absence of effects on cell viability at the whole population level (Fig 4C) suggests that a similar mechanism may also underlie TiO_2_ NF effect.

Also the haemolytic potential of the materials points to the increased biological activity of TiO_2_ NF compared to TiO_2_ NP. Interestingly, at 80 μg/cm^2^ crocidolite had significantly smaller effects than TiO_2_ NF. Haemolysis is a simple *in vitro* endpoint used to investigate the effects of the interaction between nanomaterials and biological membranes [45]. For several nanomaterials, the hemolytic activity has been considered a good predictor of pathogenicity, correlated with biological responses *in vivo* [46,47]. Surface charge is known to be a prominent driver of haemolytic activity with strongly positive particles causing perturbation and rupture of the negatively charged red blood cell membrane [48]. Both TiO_2_ NF and NP (see Table 2) displayed a positive surface charge (16 and 13.3 mV, respectively), but their haemolytic activity was markedly different. Crocidolite asbestos fibres, on the contrary, are endowed with a negative charge [49]. Thus, surface charge can explain the different haemolytic activity of crocidolite and TiO_2_ NF but not the different activity of NF and NP, which should instead be attributed to shape and to fibre length. This is also supported by the fact that the UICC crocidolite sample, which contained both long and relatively short fibres, had an average length of 2.5 ± 2.0 μm and, hence, was much shorter than the TiO_2_ NF (9.9 ± 5.8 μm).

The analysis of cellular interactions with NF by SEM and confocal microscopy showed that the incubation of macrophages with NF caused morphological changes compared to the rounded appearance of control cells. Macrophages were markedly stretched along the surface of long NF, appeared strongly deformed, and, in some cases, several cells interacted with the same fibre or bundle. Although these modifications in cell morphology may be attributed to relatively non-toxic mechanisms, such as contact migration [50] or adaptation to rough or nanostructured surfaces [51], the observed pattern is similar to that detected upon exposure of macrophages to other fibres [52,53,54] and may be indicative of attempted, partial or failed phagocytosis. Hindering of effective phagocytosis, due to the incongruity between phagocyte size and fibre length, has been observed upon exposure to other materials and related to the phenomenon of frustrated phagocytosis [12,55]. Much less evident morphological changes were observed in macrophages exposed to TiO_2_ NP, which were still rounded although with surface bulges possibly attributable to the phagocytosis of NP agglomerates [27]. The implication of this situation is likely to be impaired movement due to the presence of a long penetrating fibre, as demonstrated by others for silver nanowires [56]. Based on those results and on the possibility of frustrated phagocytosis, TiO_2_ NF are expected to affect mobility and, as such, to potentially hinder effective macrophage-mediated clearance from the lung upon *in vivo* exposure, thus favouring the development of chronic responses. Consistently, a contribution on the effects of TiO_2_ NF *in vivo* documents serious, acute and subacute cytotoxic and inflammatory effects in the lung of exposed animals [57].

In contrast with changes in cell viability, haemolytic activity, barrier competence, the inflammatory endpoints tested (NO production, *Nos2* and *Ptgs2* induction) are comparable in cells treated with NF and NP. This seems in contrast with the findings of Hamilton *et al*. [12], who noted that long, high-aspect ratio TiO_2_ nanobelts led (after LPS priming) to a significant increase in IL-1β release, while TiO_2_ NP did not. The apparent discrepancy may derive from the different endpoints investigated. Indeed, while secretion of IL-1β requires both NF-κB activation and inflammasome activity, NF-κB activation is sufficient to increase the inflammatory parameters studied here. The induction of oxidative stress is one of the paradigmatic mechanisms implied in the toxicity of TiO_2_ NP and, in particular, in their NF-κB-dependent pro-inflammatory effects [8]. Comparably small increases in ROS production were detected with EPR for both TiO_2_ NF and NP in abiotic systems. As the intrinsic production of ROS mostly depends on particle composition/surface properties, modification of shape is not, per se, expected to alter this parameter. However, this modest intrinsic reactivity did not result in a marked cell oxidative damage, as demonstrated by the lack of lipid peroxidation, although at least two distinct parameters clearly indicate the occurrence of oxidative stress in NF-treated cells were a) a limited reduction in the cellular GSH and b) a slight induction of *Hmox1*, one of the most sensitive and reliable indicators of the cell response to oxidative stress, which is linked to inflammation triggering [31]. In agreement with observations of others [58], oxidative stress also occurs in Raw 264.7 macrophages exposed to TiO_2_ NP, as demonstrated by GSH decrease and *Hmox1* induction. Given that both effective and frustrated phagocytosis lead to the generation of superoxide free radicals (O_2_^•-^) [52,59], it is likely that in macrophages exposed to either NF or NP, a limited oxidative stress ensues that triggers NF-κB-dependent induction of pro-inflammatory genes. GSH depletion resulting from exposure to TiO_2_ NF was also reported in HeLa cells by Ramkumar *et al*. [60], who used TiO_2_ NF of similar diameter and length as those used within this study. At variance with our results, Ramkumar *et al*. also reported a significant increase in lipid peroxidation and overt apoptotic changes. It should be noted, however, that the different cell models used in the two studies may well explain these divergent effects.

## Conclusions

Compared to TiO_2_ nanoparticles, TiO_2_ nanofibres cause increased cytotoxicity, haemolysis, and epithelial barrier perturbation. Distortion of cell morphology during the interaction with long fibres, similar to that seen with frustrated phagocytosis, together with marked cytotoxicity, are indicative of mechanisms that may enhance the adverse effects and ensure a sustained bio-persistence of TiO_2_ NF *in vivo*. Given the substantial similarity of TiO_2_ NF and crocidolite effects *in vitro*, the toxicity of NF *in vivo* appear worthy to be investigated, so as to properly evaluate the pertinence of adequate preventive regulatory measures.
